# Odevixibat: A Novel Bile Salt Inhibitor Treatment for Pruritus in Progressive Familial Intrahepatic Cholestasis

**DOI:** 10.7759/cureus.56886

**Published:** 2024-03-25

**Authors:** Farrah E Flattmann, Farhan S Mohiuddin, Anjuni Singh, Anamika Tandon, Stewart J Lockett, Jon D Hirsch, Chizoba N Mosieri, Adam M Kaye, Giustino Varrassi, Shahab Ahmadzadeh, Sahar Shekoohi, Alan D Kaye

**Affiliations:** 1 School of Medicine, Louisiana State University Health Sciences Center, Shreveport, USA; 2 School of Medicine, Louisiana State University Health Sciences Center, New Orleans, USA; 3 Department of Anesthesiology, Louisiana State University Health Sciences Center, Shreveport, USA; 4 Department of Pharmacy Practice, Thomas J. Long School of Pharmacy and Health Sciences University of the Pacific, Stockton, USA; 5 Department of Pain Medicine, Paolo Procacci Foundation, Rome, ITA

**Keywords:** bile salt inhibitor, pfic, familial progressive intrahepatic cholestasis, pruritus, odevixibat

## Abstract

Chronic pruritus is defined as an itch lasting greater than six weeks. It can manifest from a wide variety of etiologies, as many different substances can act as pruritogens, such as steroids, histamine, progesterone, endogenous opioids, and serotonin. In the setting of cholestatic liver disease, increased bile acids play a major role in chronic pruritus. The itching in cholestatic liver disease is worsened in intensity at night and localized frequently to the palms, soles, knees, and other pressure sites. It can be hard to manage, affecting the quality of sleep and causing irritability, poor attention, and, in some cases, depression. One such disease that results from chronic pruritus is progressive familial intrahepatic cholestasis (PFIC), a group of uncommon hereditary disorders that affects the formation of bile and its outflow from the liver. Previously, the drug ursodeoxycholic acid was used to help manage pruritus or surgical procedures, e.g., partial external biliary diversion or partial internal biliary diversion, to help control complications of the disease. This literature review will discuss three clinical studies covering the effectiveness of odevixibat in treating pruritus in patients with PFIC. Odevixibat (Bylvay) is an oral drug that has been FDA-approved to treat pruritus in patients three months of age and older with PFIC. Odevixibat prevents the reabsorption of bile salts in the intestines, resulting in decreased levels of bile salts via their excretion in stool. Several studies have determined that the drug is well tolerated and provides a nonsurgical, pharmacological treatment alternative for those with PFIC.

## Introduction and background

Chronic pruritus is an itch lasting six weeks or more [1]. It is a common manifestation of many diseases, including dermatological, allergic, infectious, and systemic diseases. Pruritus can be related to a variety of triggers, such as medication use, occupational exposures, and heat or water exposure. The sensation of an itch originates in skin-free nerve endings. Signals are transmitted through C fibers to the dorsal horn of the spinal cord, then travel to the cerebral cortex through the spinothalamic tract. Pruritus generates a spinal reflex response, the scratch [2].

The mechanism of general pruritus remains unclear, but increased concentrations of several substances such as steroids, histamine, progesterone metabolites, endogenous opioids, and serotonin have been implicated as potential pruritogens. Lysophosphatidic acid (LPA), a potent neuronal activator, was recently identified as a potential pruritogen in cholestatic patients. It was found that serum LPA concentrations were increased only in cholestatic patients who suffered from pruritus, and intradermal injections of LPA induced scratching behavior in mice in a dose-dependent manner. LPA is formed from lysophosphatidylcholine by the enzyme autotaxin (ATX). ATX activity was found to correlate with itch intensity [3] significantly. In patients with liver disease, increased bile acids may play a major role in pruritus due to impaired hepatocellular secretion, intrahepatic bile duct damage, and secondary hepatocyte secretory failure [3]. This idea is supported by findings that feeding bile salts to cholestatic patients increased pruritus, intradermal injection of bile salts caused pruritus in healthy volunteers, and binding of bile salts in the intestinal lumen by anion exchange resins ameliorates pruritus. However, the frequency and intensity of cholestatic pruritus do not correlate with the severity of cholestasis [3].

Pruritus is well known as a frequent and agonizing symptom of many types of liver diseases, particularly those with cholestatic features. The pruritus associated with cholestasis is described as an intense itching often localized to the palms and soles but can also include the knees and pressure sites. Itching is highest in intensity at night [2,3]. Scratching of the skin surface rarely alleviates the itch intensity in patients, and they often tend to rub rather than scratch. In contrast to the dermatological causes of pruritus, primary skin lesions are not detectable. However, intense scratching may lead to secondary skin lesions such as excoriations and prurigo nodularis [3].

Chronic pruritus may be mild and tolerable for some patients, but for others, it may cause profound suffering, which may involve severe skin mutilation and limit activities of daily life. Constant itching may cause distractions, which decrease concentration and impair academic and professional performance. Pruritus can also disrupt sleep and result in fatigue and psychological distress, such as depression, irritability, and even suicidal ideation [3,4]. In rare instances, intractable pruritus may become a primary indication for liver transplantation, even in the absence of liver failure, to improve the quality of life for these patients [3].

One disease with such symptoms is progressive familial intrahepatic cholestasis (PFIC), a rare autosomal recessive liver disease defined by an early onset of cholestasis with pruritus and malabsorption, which rapidly progresses and eventually culminates in liver failure. PFIC has a devastating impact not only on the lives of affected children but also on parents and families as they witness their loved ones endure terrible symptoms. Without timely intervention, such as surgery or a liver transplant, the prognosis for PFIC is bleak. A significant proportion of patients would face a drastically shortened life span, with only 50% of patients with PFIC surviving to the age of 10 years old and almost none to 20 years old [4].

## Review

Methods

This is a narrative study. We searched keywords including odevixibat, pruritus, progressive familial intrahepatic cholestasis, and PFIC on PubMed, Google Scholar, MEDLINE, and ScienceDirect sources. Sources were accessed between May 2023 and January 2024.

PFIC

PFIC is a group of uncommon genetic diseases that affect the liver and cause impaired bile flow, leading to liver damage and, eventually, liver failure. PFIC is divided into three types based on the genetic defect involved: PFIC1, PFIC2, and PFIC3 [5]. These types have distinct patterns of inheritance and are characterized by mutations in specific genes that encode proteins involved in bile formation and transport. The clinical presentation of PFIC includes pruritus, jaundice, hepatomegaly, and elevated liver enzymes [5].

PFIC is formed by mutations in genes responsible for bile acid transport and secretion, leading to impaired bile flow and an excess of toxic bile acids in the liver. Bile acids are synthesized in the liver and excreted into the bile ducts, where they manage the digestion and absorption of dietary fats [6]. In PFIC, mutations in genes such as ATP8B1, ABCB11, and ABCB4 disrupt the transport and secretion of bile acids, leading to cholestasis, or the buildup of bile acids in the liver. The excess toxic bile acids in hepatocytes cause liver damage, inflammation, and fibrosis over time, leading to progressive liver disease. PFIC is divided into three subtypes based on the affected gene and clinical presentation [6]. Type 1 PFIC is caused by mutations in the ATP8B1 gene and is characterized by low levels of phospholipids in the bile and elevated levels of serum gamma-glutamyl transpeptidase (GGT), a marker of liver damage. Type 2 PFIC is caused by mutations in the ABCB11 gene and is characterized by high levels of bile salts in the serum and urine. Type 3 PFIC is caused by mutations in the ABCB4 gene and is characterized by the accumulation of cholesterol in hepatocytes [6]. PFIC is estimated to affect one in every 50,000 to 100,000 live births. The disease is usually diagnosed in early childhood, but the age of onset and severity depend on the specific genetic mutation involved. The disease progression in PFIC is characterized by ongoing liver damage and cholestasis, leading to the development of liver fibrosis, cirrhosis, and, eventually, liver failure. The risk of liver cancer is also enhanced in individuals with PFIC, particularly in those with type 1 and type 2 PFIC [7]. The burden of PFIC on affected individuals and their families can be significant, with symptoms such as itching, fatigue, and jaundice impacting the quality of life. Treatment options for PFIC are limited, with liver transplantation being the only curative option. However, ongoing research into targeted therapies such as gene therapy and drug treatments holds promise for improving outcomes for individuals with PFIC [7].

This condition results in several complications, including cirrhosis, portal hypertension, malabsorption, jaundice, pruritus, hepatocellular carcinoma, and growth and developmental delays [7]. Chronic cholestasis caused by PFIC can lead to cirrhosis, which is characterized by fibrosis and scarring of the liver tissue. Cirrhosis caused by PFIC can also lead to portal hypertension, which is high blood pressure in the portal vein that transfers blood from the intestines to the liver. Additionally, the buildup of bile acids in the liver can impair the absorption of fat-soluble vitamins and other nutrients from the intestine, leading to malabsorption [6].

PFIC can cause jaundice, a condition in which the skin and whites of the eyes turn yellow due to the buildup of bilirubin. It can also cause severe itching or pruritus, which can be challenging to control. Furthermore, patients with PFIC may have an increased risk of developing hepatocellular carcinoma. Finally, children with PFIC may experience growth and developmental delays due to the malabsorption of nutrients and other factors related to chronic liver disease. Therefore, diagnosing and managing PFIC early is essential to avoid these complications from occurring or worsening [6].

Current treatments for PFIC

Ursodeoxycholic acid (UDCA) is a dihydroxy bile acid (3α,7β-dihydroxy-5β-cholanic acid) endogenously present in human bile, comprising 3% of bile acids. It is the primary bile acid in black bears and has been historically used in traditional Chinese medicine [8]. UDCA is orally administered in a film-coated tablet and is primarily absorbed in the small intestine through passive diffusion. Its absorption is enhanced when taken with a meal, and it is more efficacious when taken with a meal, which is related to increased biliary secretion. When UDCA is taken up into hepatocytes, it conjugates with glycine and is subsequently secreted into bile through enterohepatic circulation [8]. These conjugates are absorbed from the distal ileum through active transport. UDCA that is not absorbed reaches the colon and is converted to lithocholic acid by intestinal bacteria. Lithocholic acid is insoluble and is eliminated in the feces. The liver takes any lithocholic acid absorbed, which is sulfated and then secreted into the bile for fecal elimination [8]. UDCA exerts therapeutic effects in cholestatic liver disease through many mechanisms. In PFIC, UDCA treats the pruritus associated with PFIC. The complete or partial reduction of pruritus is seen in 35-40% of low GGT PFIC and approximately 70% of high GGT PFIC [6]. It is a safe and well-tolerated drug, and the most common adverse effect reported with UDCA use is diarrhea. Caution should be taken with the administration of UDCA in patients who are also taking bile acid resins or aluminum hydroxide due to the reduced absorption of UDCA [9].

Partial external biliary diversion (PEBD) is a surgical technique for treating PFIC and Alagille syndrome. This technique creates a jejunal conduit between the gallbladder and the abdominal wall to divert the bile flow away from enterohepatic circulation and promote drainage through a stoma. This treatment aims to reduce 50% of bile flow through the internal circulation [10]. PEBD is efficacious in improving cholestasis and has become a standard intervention for treating PFIC. The efficacy of PEBD in treating PFIC is measured through both short-term and long-term clinical outcomes. The short-term response is measured as the improvement in pruritus, and the long-term response is measured as the reduction in the need for a liver transplant [11]. Overall, PEBD has been shown to reduce serum bile acids and improve long-term outcomes for patients. However, there have been reported complications with the external stoma, and approximately 25% of patients with PFIC do not improve with PEBD [11].

Partial internal biliary diversion (PIBD) is another surgical technique, like PEBD, used to divert bile flow away from enterohepatic circulation. In contrast to PEBD, the constructed outflow tract remains in the intestinal tract, and an external stoma is not used [12]. A cholecysto-intestinal conduit is constructed within the proximal end attached to the fundus of the gallbladder, and the distal end is anastomosed to either the jejunum or ileum of the intestine. This method has been promoted over PEBD in order to avoid complications associated with the stoma and reduce malabsorption [12]. Serum levels of bile acids showed a statistically significant reduction post-operation, as did pruritus. In contrast to PEBD, there has been no reported improvement in progressive liver dysfunction with PIBD (Table 1) [12].

**Table 1:** A brief summary of current treatments, both medical and surgical, to control the symptoms of pruritus in PFIC PEBD, partial external biliary diversion; PFIC, progressive familial intrahepatic cholestasis; PIBD, partial internal biliary diversion; UDCA, ursodeoxycholic acid

Treatment	Type	Goal	Efficacy
UDCA	Medical management	Protect injured cholangiocytes, stimulate biliary secretion, detox hydrophobic bile acids, and inhibit apoptosis of hepatocytes	A complete or partial reduction of pruritus is seen in patients. It is a safe and well-tolerated drug. The most common adverse effect is diarrhea.
PEBD	Surgical	Divert bile flow away from enterohepatic circulation with the use of an external stoma	Overall, it reduces serum bile acids and improves long-term outcomes for patients. However, it can have complications with the external stoma, and approximately 25% of patients with PFIC do not improve.
PIBD	Surgical	Divert bile flow away from enterohepatic circulation without the use of an external stoma	Significant reduction in serum levels of bile acids and pruritus. Avoids complications associated with the stoma and has reduced rates of malabsorption. Contrasted to PEBD, there has been no reported improvement in progressive liver dysfunction with PIBD.

Odevixibat

Odevixibat is an oral small-molecule inhibitor of the ileal bile salt transporter. It is used to treat severe pruritus in cholestatic liver disease and PFIC [13]. Inhibition of the ileal bile salt transporter prevents the reabsorption of bile salts at the terminal ileum, reducing the serum levels of bile salts [13]. The ileal bile salt transporters function to reabsorb conjugated bile acids secreted from the liver into the intestinal tract. The conjugated bile is reabsorbed and circulated through the portal vein to the liver through enterohepatic circulation [14]. The ileal bile salt transporters preferentially reabsorb conjugated bile acids, and any bile acids that are not reabsorbed continue to circulate down to the colon and are excreted in the feces. When the reuptake of bile acids through ileal bile salt transporters is inhibited, serum levels of bile acids decrease, and bile concentration in excreted feces increases. These low levels of bile acids in the circulatory system are detected by the farnesoid X receptor (FXR), a nuclear receptor found in hepatocytes and ileal enterocytes [14]. High bile acid levels will stimulate FXR stimulation, which negatively inhibits the activity of CYP7A1, the rate-limiting enzyme of bile acid synthesis. Low levels of bile acids, as seen with ileal bile salt transporter inhibition, will decrease the activity of FXR, releasing the negative inhibition of CYP7A1 and allowing bile acid synthesis to increase [14]. The increased activity of CYP7A1 enhances the conversion of cholesterol to bile acid, increasing cholesterol formation and low-density lipoprotein (LDL) receptors’ expression on hepatocytes. More cholesterol binds to these LDL receptors, lowering serum cholesterol levels [14].

Odevixibat is available in four different doses with different formulations. Small capsules of 400 µg and 1,200 µg are swallowed, and larger capsules of 200 µg and 600 µg are opened to expose pellets that are consumed with food [15]. The recommended dose is 40 µg/kg or 120 µg/kg once a day. This small-molecule inhibitor binds to the ileal bile salt transporter reversibly and highly selectively. The high selectivity of this molecule allows it to act locally in the intestine with no systemic exposure to the drug. Oral administration of odevixibat results in minimal absorption, with the highest plasma concentrations of the drug being 0.21 and 0.62, with 40 µg/kg and 120 µg/kg doses, respectively [15]. Taking the medicine with food does not affect its absorption, and there is no accumulation of odevixibat with once-a-day administration. Over 99% of the drug is bound to plasma proteins. Odevixibat metabolism is small, with the primary method of excretion in the feces [15].

Odevixibat can affect the absorption of fat-soluble vitamins, lipophilic oral contraceptives, and other fat-soluble medications. Therefore, special considerations should be taken when patients simultaneously take drugs that fit into these categories. One study examined the adverse event profile of odevixibat and found that the most reported adverse event was diarrhea, elevated alanine aminotransferase, and elevated blood bilirubin [15].

Efficacy

Phase 3 Trial

In determining the safety and efficacy of odevixibat treatment in PFIC, 62 patients participated in a 24-week Phase 3 trial. Patients eligible for the study were PFIC1 or PFIC2 pediatric cases suffering from pruritus and increased serum bile acids at screening. The cases were randomly categorized into the control group, the experimental group, which receives odevixibat 40 μg/kg/day, or odevixibat 120 μg/kg/day. Patients were evaluated for the proportion of positive pruritus assessments (PPAs) and those with serum bile acid reduction ≥70% from baseline or ≤70 μmol/L at week 24. The proportion of PPAs investigated by caregivers using the Albireo observer-reported outcome (ObsRO) PRECISION instrument over 24 weeks [16]. Thompson et al. showed that the mean proportion of PPAs was 55% in the mixed odevixibat group compared to 30% in the control group [16]. Also, they observed a significant increase in the percentage of patients with bile acid reduction in the odevixibat experimental group compared to the control [16]. Diarrhea, or frequent bowel movements, and fever were the most common treatment-emergent adverse effects. Diarrhea or frequent bowel movements were observed in 31% of patients in the combined odevixibat group, compared to 10% of the 20 patients in the control group. Fever was also observed in 29% of the patients in the combined odevixibat group, compared to 25% of the patients in the control group. Serious adverse effects such as increased blood bilirubin, supraventricular tachycardia, increased liver function tests, dehydration, cardiac ablation, hand-foot-and-mouth disease, influenza, poor weight gain, insomnia, pruritus, dehydration, urinary tract infection, pyrexia, gastroenteritis adenovirus, H1N1 influenza, viral infection, viral upper respiratory tract infection, auricular hematoma, and neurodermatitis occurred in 7% of the cases in the combined odevixibat group compared to 10% of the patients in the control group [16]. In summary, the study determined an effective reduction in pruritus symptoms and serum bile acids compared to a control in children with PFIC and reported that odevixibat was well tolerated in these patients.

Phase 2 Trial

In determining the safety, tolerability, and efficacy of odevixibat in pediatric cases (age range one to 17 years) suffering from cholestatic liver disease and pruritus, 20 patients participated in a four-week Phase 2 study. Diagnoses included PFIC (n = 13), Alagille syndrome (n = 6), biliary atresia (n = 3), and other causes of intrahepatic cholestasis (n = 2). Patients were evaluated objectively by observing mean baseline serum bile acid level changes and subjectively with patient-reported diary data documenting pruritus and sleep. For patients included in the Phase 2 study, it has been shown that there are reductions in mean serum bile acid levels of up to 98%. Subjective patient-reported diary data in patients taking 100 μg/kg demonstrated a mean 2.8 decrease in points for pruritus (based on visual analog itch scale 0−10) and a mean 2.9 decrease in points for sleep problems (based on Patient-Oriented Scoring Atopic Dermatitis scale 0−10) [17].

No severe side effects were reported, and most adverse events observed in the study were transient. No cases of diarrhea occurred in the Phase 2 trial. Further study is warranted to determine other effects potentially associated with prolonged ileal bile acid transporter (IBAT) inhibitor use. Oral odevixibat improved serum bile acids, pruritus, and sleep disturbance in patients with PFIC. In summary, odevixibat is a potential nonsurgical option for treating the clinical signs and symptoms of PFIC and other pediatric cholestatic diseases [17].

Table 2 summarizes objective and subjective outcomes from each trial of odevixibat, as well as the safety and tolerability reported during trials. The objective outcome consists of reductions in serum bile acid levels related to the bile salt inhibitor odevixibat, which was determined to be a metric of drug efficacy, and subjective outcomes were measured by patients filling out a self-reported scale to quantify pruritus and sleep disturbances. A lower number on the scale improved the patient’s pruritus or sleep disturbances. Treatment of emergent adverse events determined safety and tolerability.

**Table 2:** Summary of outcomes and safety of odevixibat in clinical trials

Study	Population and timeline	Intervention	Objective outcome	Subjective outcome	Safety and tolerability
Phase 2 clinical trial	20 patients for four weeks	Odevixibat in an open-label study	Patients experienced an average reduction in serum bile acid levels by 47%.	Patients self-reported a significant decrease in pruritus and sleep disturbances.	No significant adverse events were reported during the trial.
Phase 3 clinical trial	62 patients for 24 weeks	Odevixibat (either 40 μg/kg or 120 μg/kg per day) or placebo in a double-blind study	Patients experienced a reduction in serum bile acid levels by 30.7% compared to the placebo group.	Patients self-reported a 25% greater improvement in pruritus compared to the placebo group.	A total of 7% of patients experienced treatment-emergent adverse events, most notably fever and diarrhea.

PFIC is a category of rare genetic disorders that are defined by impaired bile flow, leading to rapid progression to liver damage and, eventually, liver failure [18-21]. There are three types of PFIC based on the genetic defect involved: PFIC1, PFIC2, and PFIC3. These three types have distinct patterns of inheritance and are formed by mutations in specific genes that encode proteins involved in bile formation and transport [22-25]. The excess toxic bile acids in the liver can result in pruritus, jaundice, hepatomegaly, and elevated liver enzymes [26-28]. The pruritus of PFIC can cause severe cutaneous mutilation and influence routine activities due to sleep disturbance, irritability, and lack of attention. Without surgery or a liver transplant, only half of the patients will survive to age 10 [29-30]. Pharmacological treatments, including odevixbat, aim to relieve the most distressing symptom, pruritus.

## Conclusions

PFIC represents a group of rare genetic disorders characterized by impaired bile flow and rapid progression to liver deterioration and, eventually, failure. There are three types of PFIC, categorized by the genetic defect involved: PFIC1, PFIC2, and PFIC3. These three types have distinct patterns of inheritance and are caused by mutations in specific genes that encode proteins involved in bile formation and transport. The buildup of toxic bile acids in the liver can result in pruritus, jaundice, hepatomegaly, and elevated liver enzymes. Symptoms of intractable pruritus can be debilitating, leading to severe cutaneous mutilation and profound disruption of life through the loss of sleep, irritability, poor attention, and compromised academic and professional performance. Without surgery or a liver transplant, only half of the patients will survive to age 10. Pharmacological treatment aims to relieve the most distressing symptom, pruritus.

Odevixibat (Bylvay) is an orally available small-molecule inhibitor of IBAT. By blocking the reabsorption of bile salts in the terminal ileum, odevixibat decreases serum bile acid levels and reduces the symptoms of pruritus. In clinical trials, odevixibat was found to effectively reduce symptoms of pruritus and levels of serum bile acids, as well as reduce sleep disturbances in children with PFIC. It is well tolerated in patients, with most adverse effects related to GI upset, such as diarrhea, frequent bowel movements, fever, and transient elevations in transaminases. Side effects that were observed in three patients in the Phase 3 trial included increased blood bilirubin, supraventricular tachycardia, increased liver function tests, dehydration, cardiac ablation, hand-foot-and-mouth disease, influenza, poor weight gain, insomnia, pruritus, dehydration, urinary tract infection, pyrexia, gastroenteritis adenovirus, H1N1 influenza, viral infection, viral upper respiratory tract infection, auricular hematoma, and neurodermatitis. Overall, odevixibat is a nonsurgical pharmacological treatment alternative for patients with PFIC. By attenuating severe pruritus and reducing serum bile acid levels, odevixibat may reduce or delay the need for diversion surgery and mitigate associated surgical risks. It may offer a tangible improvement in the quality of life for those with PFIC suffering from chronic pruritus.
